# A Small Epitope Tagging on the C-Terminus of a Target Protein Requires Extra Amino Acids to Enhance the Immune Responses of the Corresponding Antibody

**DOI:** 10.4014/jmb.2401.01036

**Published:** 2024-05-13

**Authors:** Kyungha Lee, Man-Ho Cho, Mi-Ju Kim, Seong-Hee Bhoo

**Affiliations:** 1 Graduate School of Biotechnology, Kyung Hee University, Yongin 17104, Republic of Korea; 2 Department of Genetics and Biotechnology, Kyung Hee University, Yongin 17104, Republic of Korea; 3 Institute of Life Sciences & Resources and Department of Food Science and Biotechnology, Kyung Hee University, Yongin 17104, Republic of Korea; 4 Graduate School of Green-Bio Science, Kyung Hee University, Yongin 17104, Republic of Korea

**Keywords:** 2B8 peptide, peptide epitope, epitope tagging system, antibody

## Abstract

Protein-specific antibodies are essential for various aspects of protein research, including detection, purification, and characterization. When specific antibodies are unavailable, protein tagging is a useful alternative. Small epitope tags, typically less than 10 amino acids, are widely used in protein research due to the simple modification through PCR and reduced impact on the target protein's function compared to larger tags. The 2B8 epitope tag (RDPLPFFPP), reported by us in a previous study, has high specificity and sensitivity to the corresponding antibody. However, when attached to the C-terminus of the target protein in immunoprecipitation experiments, we observed a decrease in detection signal with reduced immunity and low protein recovery. This phenomenon was not unique to 2B8 and was also observed with the commercially available Myc tag. Our study revealed that C-terminal tagging of small epitope tags requires the addition of more than one extra amino acid to enhance (restore) antibody immunities. Moreover, among the amino acids we tested, serine was the best for the 2B8 tag. Our findings demonstrated that the interaction between a small epitope and a corresponding paratope of an antibody requires an extra amino acid at the C-terminus of the epitope. This result is important for researchers planning studies on target proteins using small epitope tags.

## Introduction

When the target protein-specific antibody is absent, tagging is an essential tool in many biochemical experiments, such as protein purification, identification, quantification, and localization. Several epitope tags have been developed for various experimental purposes since the first use of small polypeptides as epitope tags for purification of recombinant proteins [1]. A cloning target gene with tags can be achieved by a relatively simple PCR method with primers containing the tag sequence or insertion into vector DNA containing the tag sequence [2, 3]. Therefore, shorter tagging is preferred to handle cloning.

When selecting a suitable tag in a protein experiment, the effects of the added tag sequence on the target protein should be carefully considered. Regarding the size and chemical properties of the tags, small epitope tags, such as FLAG (DYKDDDDK) [4], 6 X His (HHHHHH) [5], HA (YPYDVPDYA) [6, 7], and c-Myc (EQKLISEEDL) [8, 9], have often been preferred for experiments. These small epitope tags are advantageous over the larger protein tags, such as glutathione S-transferase (GST) or maltose-binding protein (MBP), in the process of cloning and the possible effects on the tagged target proteins. In addition, tagging location should also be carefully considered because tagged additional amino acid sequences can change the 3-D structures or functions of the tagged target protein [10, 11]. A tag should not be buried in the structural core of the protein or near the binding domain where it could interfere with the binding of the real binding partners [12, 13]. Therefore, most researchers add tagged sequences on the side of the N- or C-terminal ends of the target proteins to minimize their 3-D folding of the main sequence during translation of the protein [14].

Our previous study reported a short peptide epitope sequence, “RDPLPFFPP,” identified from antibody generation, followed by epitope mapping of the antigen protein bacteriophytochrome of *Deinococcus radiodurans* [15, 16]. This epitope sequence, named 2B8, was unique because it was not found in any known protein database. This uniqueness means that the antibody recognizing the 2B8 epitope can have higher specificity to the epitope. The binding affinity of the antibody to the 2B8 epitope also showed promise as a tagging candidate, with an extremely low K_d_ value in the picomolar range (10^-12^) (Fig. S1) compared to most commercial tags, such as HA, Flag, and Myc, which have high affinity K_d_ values of 4.5 nM, 6.5 nM, and 80 nM, respectively [17, 18].

However, we noticed during the protein tagging experiments that the tagged 2B8 epitope at the C-terminus of the GFP (green fluorescence protein) showed a highly decreased immune response with a corresponding antibody, unlike tagging at the N-terminus. This phenomenon was also identified with a widely used commercial Myc tag.

In this study, we analyzed the effects of amino acids around the epitope sequence to determine the reason for the decreased immune response for C-terminus tagging. We noticed that the 2B8 and Myc epitopes need additional amino acids at the C-terminus for proper binding with corresponding antibodies. This result is important for researchers working with tagged proteins to enhance the stability and functionality of proteins using improved C-terminal tagging.

## Materials and Methods

### Expression of Epitope-Tagged GFP Proteins

All DNA constructs of 2B8- and Myc-tagged GFP proteins produced by PCR were cloned into a pET-21(a) expression vector and expressed in *Escherichia coli* (*E. coli*) strain BL-21(DE3) cells. Transformed cells with each construct of expression vector were grown in LB medium containing 100 μg/ml of ampicillin at 37°C until the optical density at 600 nm (OD600) was 0.4–0.6. The final concentration of 0.5 mM isopropyl β-D-1-thiogalactopyranoside (IPTG) was added into the culture medium to induce the tagged target protein expression at 25°C for 16 h. For protein extraction and purification, cells were harvested by centrifugation (5,000 ×*g* for 5 min) and lysed by sonication in PBS. The lysates were then centrifuged at 10,000 ×*g* for 20 min to obtain a total protein solution containing expressed target proteins.

### Adjustment of Tagged GFP Protein Quantities

To adjust the concentration of each tagged GFP protein, the absorbances at Ex-398 nm were measured for protein samples containing 2B8-tagged GFP [19], where only GFP protein exhibits its maximum absorption (Fig. S2) and exhibits fluorescence at Em-510 nm [20]. After the initial measurement, the concentration of all tagged GFP proteins was adjusted by dilution to achieve similar fluorescence values in all samples. All these standardized samples were used in subsequent experiments.

### Western Blot and Dot Blot Analyses

The protein samples were mixed with sample buffer [125 mM Tris-HCl (pH 6.8) containing 4% (w/v) SDS, 0.005% (w/v) bromophenol blue, 20% (v/v) glycerol, 5% (v/v) β-mercaptoethanol] for SDS-PAGE. The prepared samples were separated in 12% polyacrylamide gels and then transferred to a polyvinylidene difluoride (PVDF) membrane (Invitrogen, USA). Anti-GFP antibody (Santa Cruz, USA) and anti-2B8 specific antibody (Biojane Co., Ltd., Republic of Korea) or anti-Myc specific antibody (Santa Cruz) were used as primary antibodies to detect target protein. Horseradish peroxidase-conjugated anti-mouse IgG was used as a secondary antibody. Western blot signals were detected by chemiluminescence using the ECL reaction (GE Healthcare, UK). Dot blot analysis was performed to test the detection of tagged protein in native conditions. Each protein sample was loaded onto a nitrocellulose membrane, and followed the identical process of western blot analysis from the blocking to detection step.

### Immunoprecipitation (IP) Assay and Peptide Elution

Immunoprecipitation (IP) experiments were conducted using anti-2B8, anti-Myc, and anti-GFP specific antibodies. Each tag-specific antibody was mixed with the tagged GFP target protein solution and incubated at 4°C for 1 h with shaking. Subsequently, protein G-agarose beads (Thermo Fisher Scientific, USA) were added to the mixtures of the antibody and the protein, and then incubated for at least 1 h at 4°C with gentle shaking. The immunoprecipitation mixtures were then centrifuged to remove the supernatant. Precipitated beads were washed with five-bead volumes of PBS containing 0.05% Tween 20.

To elute the 2B8- or Myc-tagged GFP target protein from the bound beads, a peptide solution was added to the immunoprecipitated bead mixtures. This mixture was incubated for 5-10 min with gentle pipetting. Eluted target proteins were obtained from the supernatants after centrifugation. The immunoprecipitated target proteins and the eluted purified target proteins were confirmed by western blot analysis. The anti-GFP antibody was used as the primary antibody for detection to avoid discrepancies in affinities between different tags and their corresponding antibodies.

## Results

### 2B8 Epitope Tagging Showed Different Detection Intensities to the Tagging Position

**Fig. 1:**
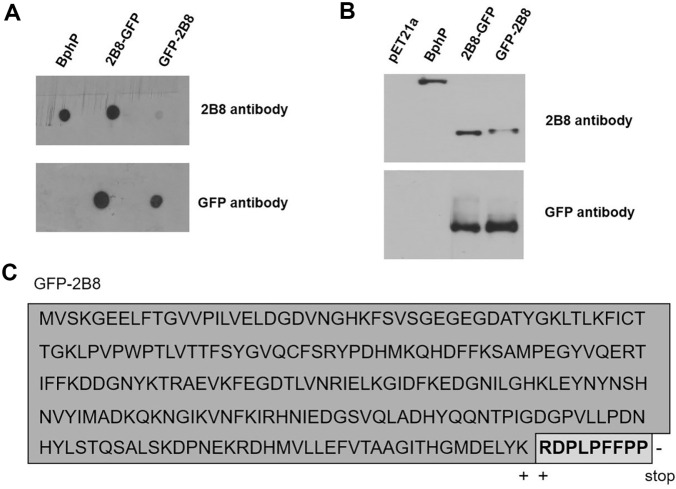
Analysis of 2B8-tagged GFP proteins. Dot blot (**A**) and western blot (**B**) analyses using anti-2B8 antibody and anti-GFP antibody. (**C**) Sequence of GFP with C-terminal 2B8 tag.

The C-terminal tagging of the 2B8 tag of any target protein was problematic for several years in our lab due to its low detection intensity. To determine the reason for the immune response discrepancy regarding the anti-2B8 antibody to the 2B8 epitope tag, various modified 2B8 epitopes were analyzed by tagging at the N-terminal (2B8-GFP) and C-terminal (GFP-2B8) positions of GFP protein. To substantiate the binding efficacy, the original protein bacterial phytochrome photoreceptor (BphP) [15], from which the sequence of the 2B8 tag is derived (Fig. S3), was also expressed and tested in dot blot (Fig. 1A) and western blot (Fig. 1B) analyses. Both original antigen BphP and the 2B8-GFP exhibited detection signals against the anti-2B8 specific antibody. On the other hand, the GFP-2B8 revealed significantly decreased detection intensity of the anti-2B8 specific antibody. However, the dot blot and western blot analyses against GFP protein with an anti-GFP specific antibody showed similar detection signals for the two tagging positions, confirming that the detection variation is not due to differences in the amount of target protein. This detection signal difference was observed consistently in both the dot blot and western blot analyses, which suggests that this phenomenon is not due to the structure of the tagged target proteins.

As a first step, we focused on the proximity effect of the 2B8 tag sequence. Since the last amino acid of the GFP protein was lysine (K, +charge) and the connected first amino acid of the 2B8 tag was arginine (R, +charge)(Fig. 1C), we assessed possible electrostatic charge effects [21] for interaction between the paratope of an antibody and the epitope. Various modified sequences of the 2B8 tag were designed to explore these effects.

### Effects of Neighbor Amino Acids on the C-Terminal Tagged 2B8

**Fig. 2:**
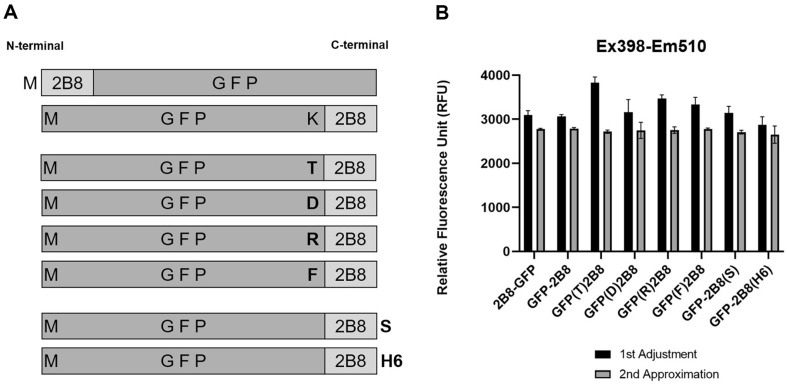
Amino acid modification of 2B8-tagged GFP proteins. Scheme of modified 2B8-tagged GFPs. Quantification of modified 2B8-tagged GFPs. All proteins were measured for fluorescence at Ex 398-Em510 nm.

To verify whether the decreased detection of the C-terminus tagged 2B8 was due to the charge effect of the last amino acid K of the GFP protein, the last amino acid of the target GFP protein was changed (Fig. 2A). The positively charged last amino acid K of GFP was substituted to verify the possible effects of charges, polarity, and hydrophobicity. The K of GFP was substituted with R (larger same + charge), T (polar), D (- charge), or F (hydrophobic) amino acids. In addition, we tried adding extra amino acids on the terminal end of the 2B8 tag to assess the impact on the “stop” of the C-terminal end. All the expressed 2B8-tagged GFP protein samples were adjusted to make them similar to the protein concentration based on fluorescence values to minimize the error from the protein quantities (Fig. 2B).

**Fig. 3:**
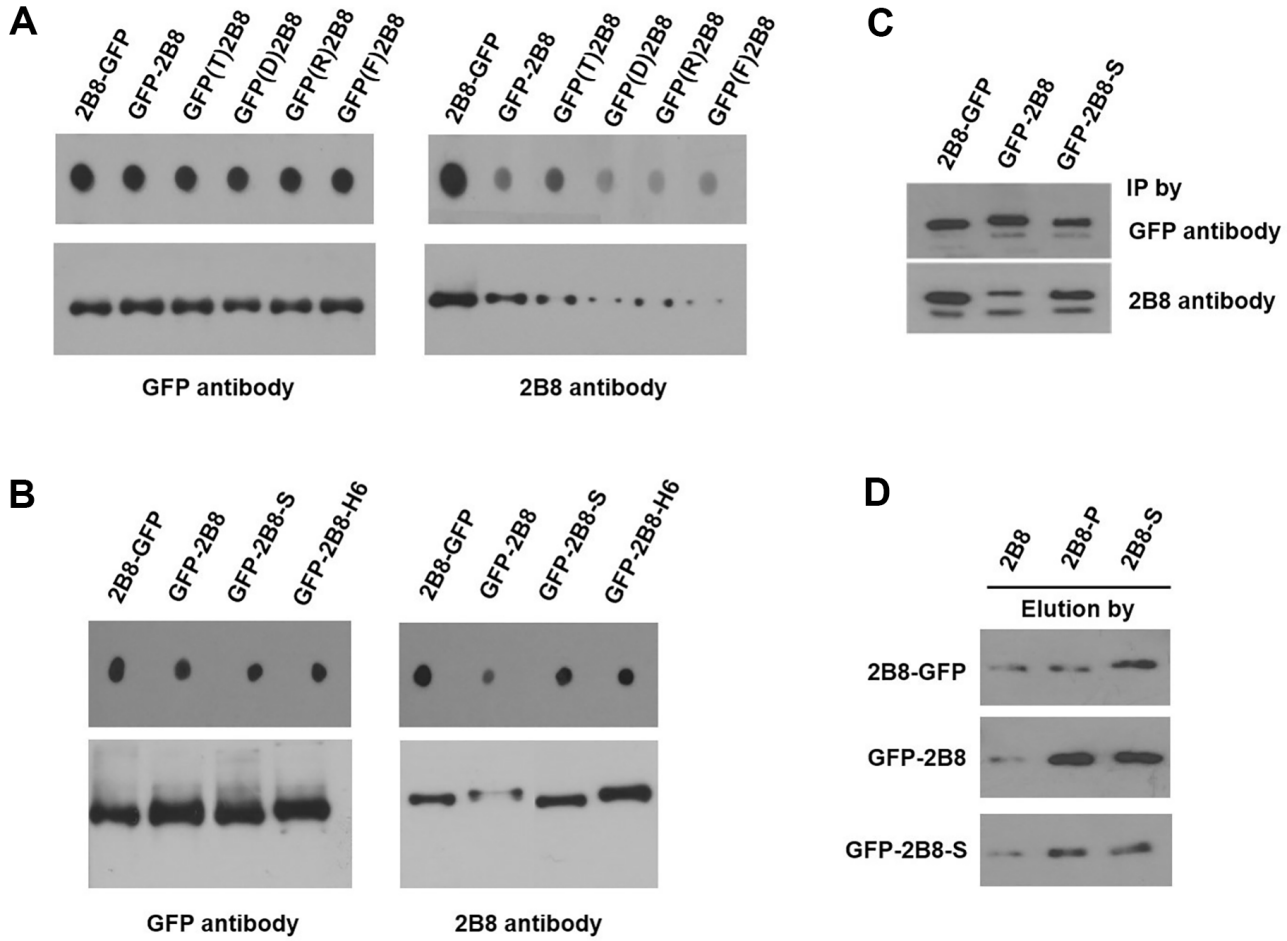
Analysis of 2B8-tagged GFPs with adjacent amino acid modification. Dot blot (top) and western blot (bottom) analyses of amino acid-modified GFPs fused with 2B8 tag. Dot blot and western blot were conducted using anti-GFP antibody and anti-2B8 antibody. Dot blot (top) and western blot (bottom) analyses of 2B8-tagged GFP with extra amino acid addition. Anti-GFP antibody and anti-2B8 antibody were used in the analyses. Immunoprecipitation (IP) analysis of anti-GFP antibody or anti-2B8 antibody. The western blot analysis for confirmation of precipitated protein was conducted using an anti- GFP antibody. Peptide elution after IP using anti-2B8 antibody. Western blot to confirm the eluted protein was performed using an anti-GFP antibody.

Dot blot and western blot assays were carried out for the 2B8-GFP, GFP-2B8, and the C-terminus tagged modified GFP-(T, D, R, F)-2B8 (Fig. 3A). Even though all the tested 2B8- tagged GFP samples were detected with the same intensities with an anti-GFP specific antibody, the C-terminus tagged modified GFP-(T, D, R, and F)-2B8 proteins showed lower detection signals with the anti-2B8 specific antibody. Therefore, the decreased detection of the C-terminus tagged 2B8 epitope is not due to the effects of charge, polarity, or hydrophobicity of the preceding amino acid of the 2B8 tag. However, C-terminal 2B8 with an additional serine or His-tag exhibited restored detection signals comparable to that of N-terminus tagged 2B8 (Fig. 3B). This result suggests that the anti-2B8 specific antibody requires an extra amino acid at the C-terminal end. The antibody showed no specific preference for several extra amino acids tested (data not shown). It means that the extra amino acid is working as a platform rather than a real specific epitope.

### Immunoprecipitation (IP) Assay to Confirm the Extra Amino Acid of C-Terminus Tagged 2B8

Immunoprecipitation (IP) is a commonly used biochemical tool for protein research techniques, such as protein purification and protein-protein interactions. IP was conducted to validate the effectiveness of adding extra amino acids to the C-terminus of the 2B8 tag (Fig. 3C). In advance, three constructs were immunoprecipitated with an anti-GFP specific antibody to confirm that all tested proteins started with similar concentrations. As expected, in the IP experiment using an anti-2B8 specific antibody, the immune response to the C-terminus 2B8 tag containing the extra amino acid was completely recovered. Compared to 2B8-GFP and GFP-2B8, the GFP-2B8-S showed similar IP results to 2B8-GFP. This result is consistent with the western blot results confirming that the decreased detection of C-terminus tagging of the 2B8 tag can be overcome with extra amino acids.

We also tested whether a 2B8 peptide with an extra C-terminus amino acid can affect the purification of a 2B8-tagged target protein during immunoprecipitation. Generally, immunoprecipitated proteins can be purified by elution from the IP beads using the same sequence of epitope peptide. An extra amino acid-added 2B8-S peptide showed increased elution compared to the original 2B8 peptide (Fig. 3D). Despite different amounts of the three proteins 2B8-GFP, GFP-2B8, and GFP-2B8-S precipitated by the anti-2B8 specific antibody, the amount of eluted 2B8-tagged proteins increased when using the amino acid-added peptides, 2B8-P and 2B8-S, compared to the original. These IP and peptide elution results confirmed that additional amino acids are required on the C-terminal side of the 2B8 tag for binding of the anti-2B8 specific antibody.

### Comparison of 2B8 Epitope Results with a Myc Tag

**Fig. 4:**
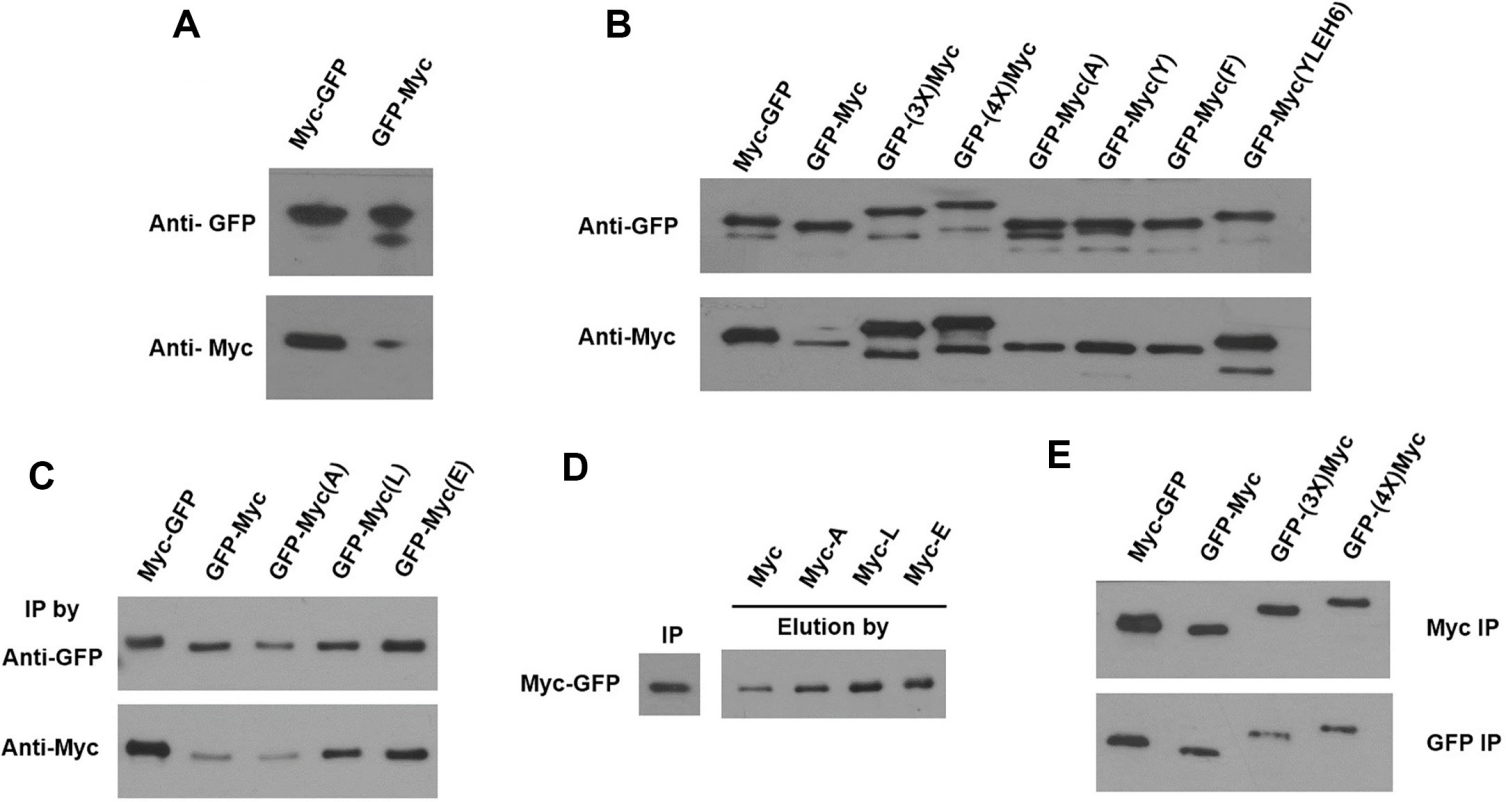
Immunity analysis of the Myc-tagged GFP proteins. Western blot analysis of GFP fused with a Myc tag at the Nor C-terminal using anti-GFP antibody and anti-Myc antibody. Western blot analysis of GFP-Myc with amino acid or multi-tag extension. Immunoprecipitation (IP) analysis of amino acid-added GFP-myc. Anti-GFP antibody or anti-Myc antibody was used in IP, and the western blot analysis for confirmation of precipitated protein was conducted using an anti-GFP antibody. Peptide elution of Myc-GFP after IP using anti-Myc antibody. Western blot to confirm the eluted protein was performed using an anti-GFP antibody. IP analysis of multi-Myc tagged GFP at the C-terminal. Eluted proteins were confirmed by western blot using anti-GFP antibody.

The result that a small epitope tag needs extra amino acids when fused at the C-terminus was unexpected. We often have experienced a similar problem with commercial epitope tags such as Myc. To overcome this weakness of detection, many researchers attach multiple repeated tags in the C-terminus of the target proteins. To assess whether this limitation is unique to the 2B8 epitope or a general phenomenon of small epitope tags, tests performed on 2B8 were repeated with a commercial Myc tag. Surprisingly, similar results to the 2B8 tag were obtained with the Myc tag epitope; GFP-Myc showed a significantly lower detection signal compared to the Myc-GFP in a western blot using an anti-Myc specific antibody (Fig. 4A).

To verify whether these results are consistent, C-terminus tagged Myc epitopes were modified by adding an extra amino acid and tagging conventional repeat tags. The results were very similar to those of the 2B8 tag; the Myc tag attached to the C-terminus with extra amino acid(s) restored the detection signal as with the N-terminus tagged protein (Fig. 4B). When multiple amino acids or multiple Myc tags were added in the C-terminus, compared to adding a single amino acid, the detection signals increased slightly but not significantly. This result demonstrates that small epitope tags need at least one or more extra residues for proper docking in the C-terminal end to achieve interactions between the epitopes and paratopes of corresponding antibodies. Collectively, the reduced detection signal of a C-terminus tagged Myc tag can be overcome by adding amino acids, and the repeated multiple Myc tagging is not required to increase the detection signals.

### Immunoprecipitation (IP) and Peptide Elution of Myc-Tagged Protein

Since we noticed the recovered detection in the western blot analysis for the C-terminal tagged Myc with extra amino acids, we needed to confirm whether this result was consistent with the native protein in IP experiments. Myc-GFP, GFP-Myc, and single amino acid (A, L, E)-added GFP-Myc proteins were immunoprecipitated using anti-GFP specific or anti-Myc specific antibodies. All samples were precipitated similarly by the anti-GFP specific antibodies (Fig. 4C). In contrast, different results were observed in IP samples with the anti-Myc specific antibodies. Compared to the Myc-GFP protein, the precipitated protein of GFP-Myc significantly decreased. However, GFP-Myc (L) and GFP-Myc (E) with a single amino acid added recovered to the level of Myc-GFP, except GFP-Myc (A), probably because the alanine is too small to support the interaction of the paratope of the Myc antibody (Fig. 4C).

The need for an extra amino acid was confirmed by modified Myc peptides for elution of immunoprecipitated Myc-tagged GFP proteins. Immunoprecipitated Myc-GFP proteins by the anti-Myc specific antibodies were eluted using the original Myc peptide or a Myc peptide with a single amino acid (A, L, or E) added at the C-terminal end. The results showed more proteins were eluted from the Myc-A, Myc-L, and Myc-E peptides (Fig. 4D). The consistent pattern revealed that the results from the 2B8 epitope tag reflect a general phenomenon for small epitope tags. These results demonstrate the need for additional amino acids of a C-terminus small epitope tag, which increases target protein detection and purification.

## Discussion

Functional studies of proteins are important aspects of research in life science. The prerequisite of protein research is detection and purification of the target protein. Various biochemical tools such as leveling and tagging have been developed for this purpose. The target protein-specific antibody is the best way to study the target protein. However, protein tagging is a useful alternative if there is no target protein-specific antibody available. Even though several protein tags have been developed, small epitope tags that use small peptides as an epitope of the target protein are widely used [22]. This system allows easy modification and enables detection and purification of the target protein without the production of antibodies specific to the target protein [23-25]. However, problems such as non-specific detection or unexpected interactions in protein experiments are reported for small epitope tags [26, 27].

The 2B8 epitope identified from antibody generation with a bacteriophytochrome as an antigen has a unique amino acid sequence. The 2B8 epitope sequence (RDPLPFFPP) has no copy in any reported protein, suggesting that the antibody to this epitope is very specific to the 2B8 epitope [16, 28]. Results of biochemical experiments using the 2B8 epitope showed highly specific and strong detection signals of 2B8-tagged target proteins by the 2B8-specific antibody. Epitope tags are most often attached to the C-terminus of the target proteins, probably due to the possible conformation change of the target protein by the N-terminus tagged extra amino acids during protein translation [29]. However, we accidentally noticed that the C-terminus tagged 2B8 epitope exhibited significantly decreased antibody binding reactivity. This problem was observed with both 2B8 and the commercial Myc tag. Therefore, we decided to investigate the reason for this phenomenon. Initially, we focused on the influence of the neighbor amino acids of epitope tags because the binding of an epitope requires proper interactions with the paratope of the antibody. If the amino acid neighboring the epitope sequence produces unexpected charge, polarity, or hydrophobicity changes, binding of the antibody will be decreased; however, this was not the reason for the problem (Fig. 3A). After several systematic trials with changes, we noted that the C-terminus tagged small epitope needed one or more extra amino acids on the C-terminal end to detect the epitope (Figs. 3B and 4B). There were no preferred amino acids that could be the tag for recovery of immunity of the C-terminus tagged epitopes. Even the GFP-Myc(A) failed to recover the decreased immunity of C-terminus tagged GFP-Myc (Fig. 4C). Since alanine is the smallest amino acid, interactions between the epitope and the paratope of the antibody further require a minimum volume of the amino acid side chain to achieve complete interaction and full immunity. We assume the small epitope sequence itself is not enough to interact with the paratope of the antibody. Small epitopes need an extra amino acid as a platform on the C-terminal side, and in the case of N-terminus tagged epitopes, acquire the extra platform amino acids automated by the following target proteins.

Multi-tagging is frequently used to enhance the detection of a target protein by increasing the number of corresponding antibodies that bind to the tag [30, 31]. However, multi-tagged GFP-(3X)Myc and GFP-(4X)Myc did not show 3-4 times increased immunity compared to the Myc tags of GFP-Myc(YLEH6), GFP-Myc(Y), and d -Myc(F) in our experiments (Fig. 4B). Here, we expect that the increased detection sensitivity of multi-tagged Myc compared to the single Myc tag is probably due to the increased immunity imparted by extra amino acids automatically added to the Myc epitopes by multiplication rather than being solely attributed to simple quantitative factors.

Additionally, multi-tagging of Myc epitope is not as efficient as expected for Myc-tagged target protein purification by IP. Moreover, IP of Myc-GFP, GFP-Myc, GFP-(3X)Myc, and GFP-(4X)Myc using the anti-GFP specific antibody yielded a slight reduction in precipitated proteins compared to the single-tagged GFP-Myc (Fig. 4E). Therefore, multi-tagging of highly charged Myc tags needs to be considered carefully because the Myc epitope contains a positively charged K and four negatively charged amino acids (three E and one D). Multiplication of Myc tagging results in the inclusion of many unexpected charges on the target protein, which can interfere with its function or structure. If the target protein is tagged with the Myc epitope, the addition of a few amino acids to the C-terminal end would be enough.

In this study, we report an important new finding about small epitope tags in which the decreased immunity of C-terminus tagged small epitopes such as 2B8 and Myc recovered their immunity through addition of extra amino acids to the C-terminal end. We think this phenomenon is the “platform” on which the epitopes need at least one additional sequence to interact with the paratope of the corresponding antibody for full immunity. The sequences of most small epitope tags were identified by epitope mapping with a C-terminus extended peptide condition. Since the amino acid following the epitope was not specific to the immunity, investigators ignored this extra residue in the real epitope sequence. However, the paratope of the antibody recognizes the minimum size of the platform residues of the epitope and extra amino acids in the C-terminus to complete the platform residues. We believe our findings provide important information in the field of biochemistry, especially to those in the field of protein research.

## Supplemental Materials

Supplementary data for this paper are available on-line only at http://jmb.or.kr.
