# Supplementary material for: A Small Epitope Tagging on the C-Terminus of a Target Protein Requires Extra Amino Acids to Enhance the Immune Responses of the Corresponding Antibody
Source: J Microbiol Biotechnol. 2024 May 13;34(6):1222–8. doi: 10.4014/jmb.2401.01036 (PMC11239440; doi:10.4014/jmb.2401.01036)
Supplement: Supplementary file 1 [file jmb-34-6-1222-supple.pdf]

Advanced Kinetics

Experiment: 2B8 20190322
Description:
Date: Friday, March 22, 2019
Time: 9:36:03 PM

Run Settings

	Step Type	Duration (s)	Position
1	Initial Baseline	180	Tube
2	Custom	20	Tube
3	Baseline	20	Tube
4	Custom	20	Tube
5	Baseline	20	Tube
6	Custom	20	Tube
7	Baseline	20	Tube
8	Baseline	300	Tube
9	Loading	600	Drop
10	Baseline	180	Tube
11	Association	600	Tube
12	Dissociation	600	Tube

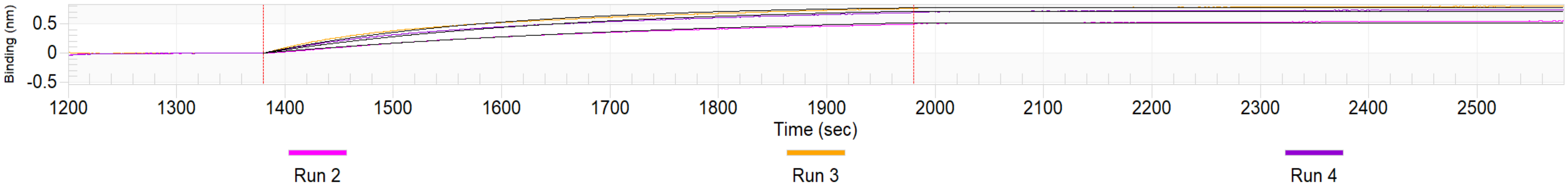

Index	Sample ID	Conc. (nM)	Information	KD (M)	ka (1/Ms)	ka Error	kd (1/s)	kd Error	Rmax	Rmax Error	R equilibrium	X^2	R^2
1	BphP ref	0											
2	BphP _5ug/ml	66.67		<1e-12	3.506e4	2.681e2	<1e-7		0.6908	0.007327	0.6908	0.6424	0.9921
3	BphP _10ug/ml	133.3		<1e-12	3.506e4	2.681e2	<1e-7		0.826	0.003997	0.826	0.6424	0.9921
4	BphP _7.5ug/ml	100		<1e-12	3.506e4	2.681e2	<1e-7		0.8188	0.006104	0.8188	0.6424	0.9921

Fig.S1 Kinetic analysis of 2B8 epitope of BphP and the anti-2B8 antibody.

Kinetics was analyzed by the BLI(bio-layer interferometry) technology by the BLItz system (Fortebio, USA). The 2B8 showed a picomolar level of Kd value (Red box).

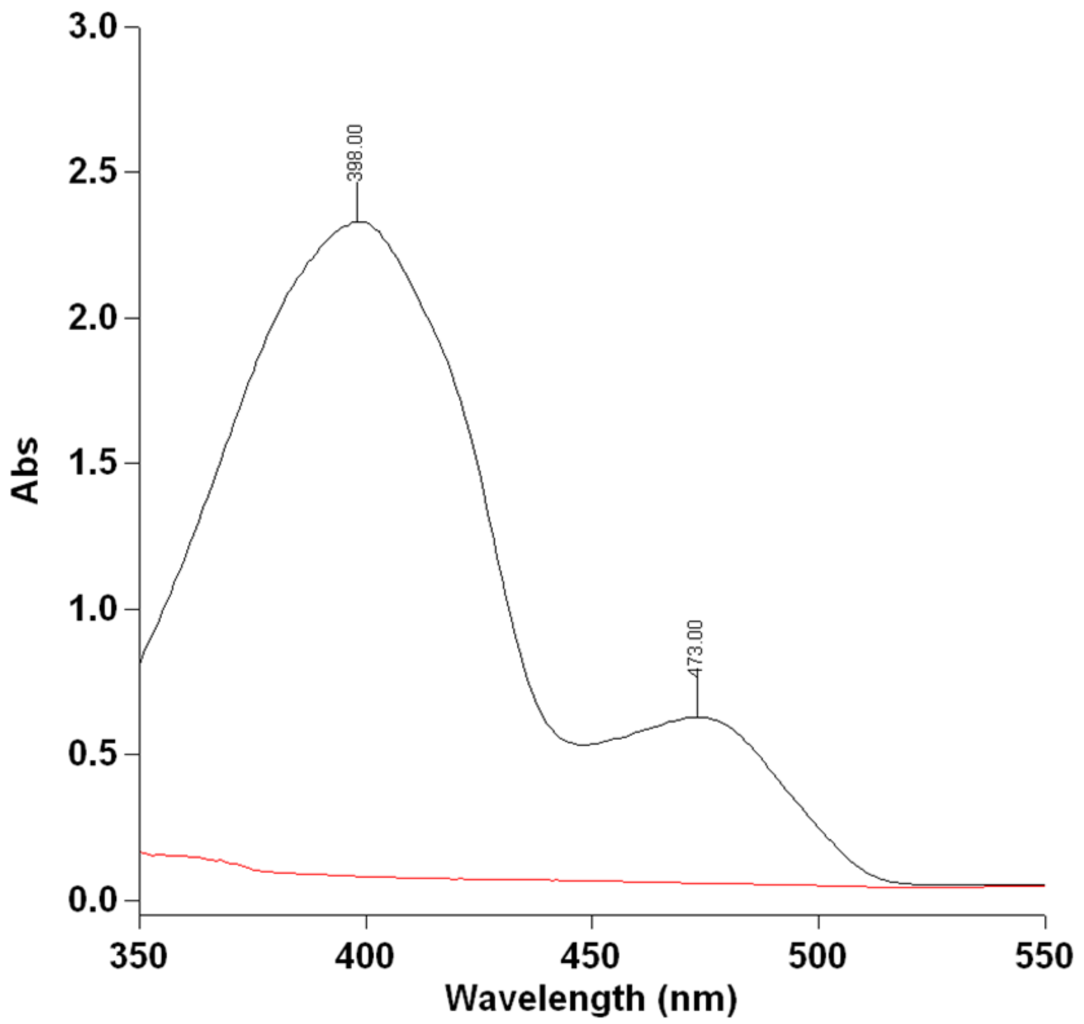

Fig.S2 Absorbance scan of GFP.

The absorption spectrum of the GFP protein used in the experiment was scanned, showing the maximum absorbance peak at 398 nm.

2B8 sequence

MSRDPLPFFPPLYLGGPEITTENCEREPIHIPGSIQPHGALLTADGH
SGEVLQMSLNAATFLGQEPTVLRGQTLAALLPEQWPALQAALPPG
CPDALQYRATLDWPAAGHLSLTVHRVGELLILEFEPTEAWDSTGPH
ALRNAMFALESAPNLRALAEVATQTVRELTGFDRVMLYKFAPDATG
EVIAEARREGLHAFLGHRFPASDIPAQARALYTRHLLRLTADTRAAA
VPLDPVLNPQTNAPTPLGGAVLRATSPMHMQYLRNMGVGSSLSVS
VVVGGQLWGLIACHHQTPYVLPPDLRTTLEYLGRLLSLQVQVKEAA
DVAAFRQSLREHHARVALAAAHSLSPHDTLSDPALDLLGLMRAGGL
ILRFEGRWQTLGEVPPAPAVDALLAWLETQPGALVQTDALGQLWPA
GADLAPSAAGLLAISVGEGWSECLVWLRPELRLEVAWGGATPDQA
KDDLGPRHSFDTYLEEKRGYAEPWHPGEIEEAQDLRDTLTGALGE
RLSVIRDLNRALTQSNAEWRQYGFVISHHMQEPVRLISQFAELLTR
QPRAQDGSPDSPQTERITGFLLRETSRLRSLTQDLHTYTALLSAPP
PVRRPTPLGRVVDDVLQDLEPRIADTGASIEVAPELPVIAADAGLLR
DLLLHLIGNALTFGGPEPRIAVRTERQGAGWSIAVSDQGAGIAPEYQ
ERIFLLFQRLGSLDEALGNGLGLPLCRKIAELHGGTLTVESAPGEGS
TFRCWLPDAGPLPGAADA

Fig.S3 Sequence of BphP.

Sequence of BphP(Bacterial phytochrome photoreceptor) from which the 2B8 epitope tag was derived. The BphP contains the 2B8 tag sequence at the N-terminal side.
